# Supplementary material for: Ex Vivo Assessment of Tumor-Targeting Fluorescent Tracers for Image-Guided Surgery
Source: Cancers (Basel). 2020 Apr 17;12(4):987. doi: 10.3390/cancers12040987 (PMC7226456; doi:10.3390/cancers12040987)
Supplement: Supplementary file 1 [file cancers-12-00987-s001.pdf]

Supplementary Materials: Ex Vivo Assessment of Tumor-Targeting Fluorescent Tracers for Image-Guided Surgery

Fortuné M.K. Elekonawo, Jan Marie de Gooyer, Desirée L. Bos, David M. Goldenberg, Otto C. Boerman, Lodewijk A.A. Brosens, Andreas J.A. Bremers, Johannes H.W. de Wilt and Mark Rijpkema

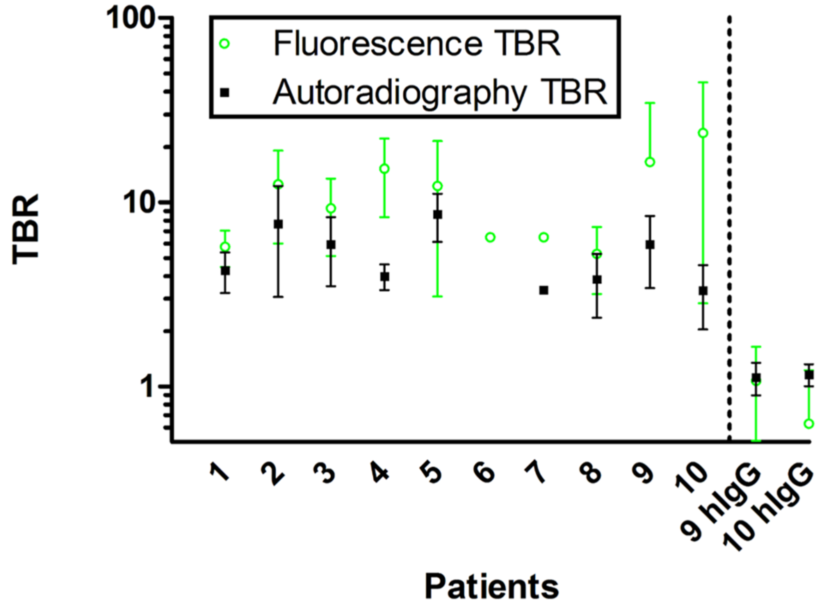

Figure S1. Tumor-to-background ratio for fluorescence and autoradiography of tissue incubated with 111In-DOTA-hMN14-IRdye800CW per patient. Tissue of patient 9 and 10 was split and also incubated with the control antibody conjugate, 111In-DOTA-hIgG-IRDye800CW. The autoradiography of patient 6 is missing due to failure of the radiolabeling.

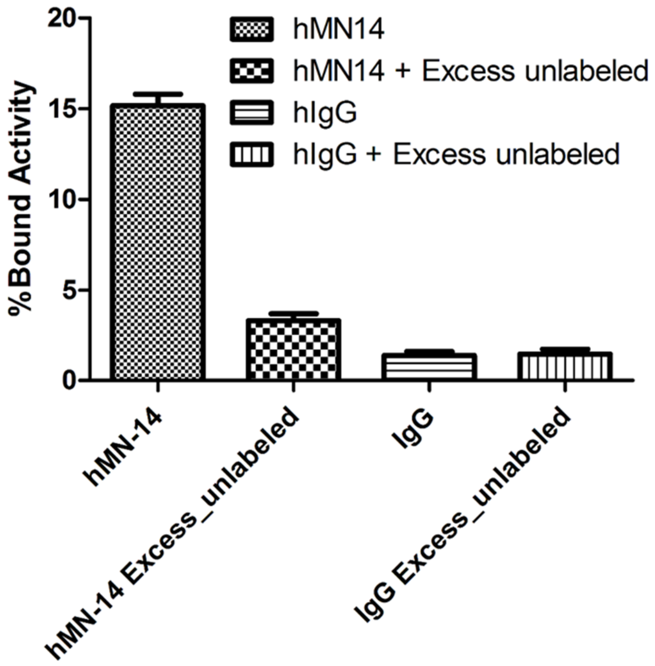

Figure S2. Binding assay with CEA-positive LS174T cells using dual-labeled hMN-14 and dual-labeled hIgG (control antibody).

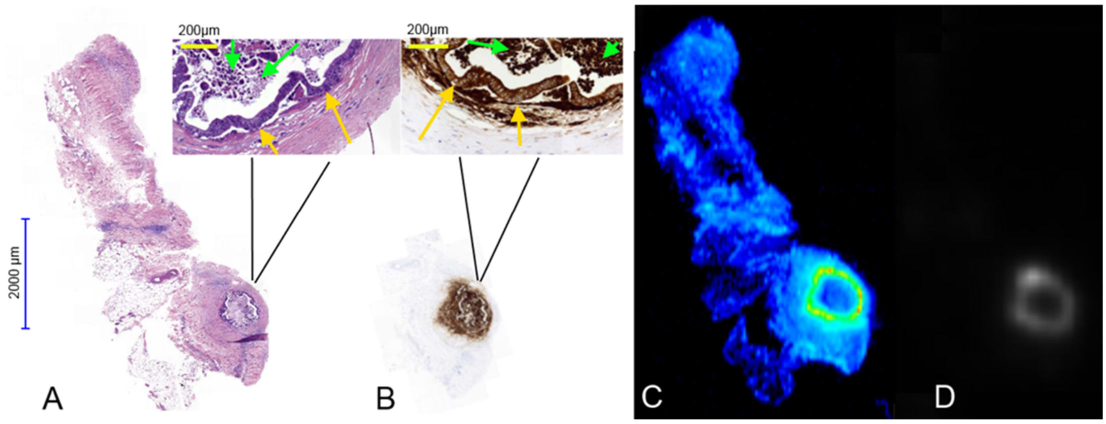

Figure S3a: Tumor with a necrotic core (other patient than depicted in figure 3b). A. H&E staining and zoom, B. CEA immunohistochemical staining, C. Flatbed fluorescence scan image, D. Autoradiography. Note the tracer uptake in the viable tumor border (yellow arrows) and the necrotic tumor core (green arrows).

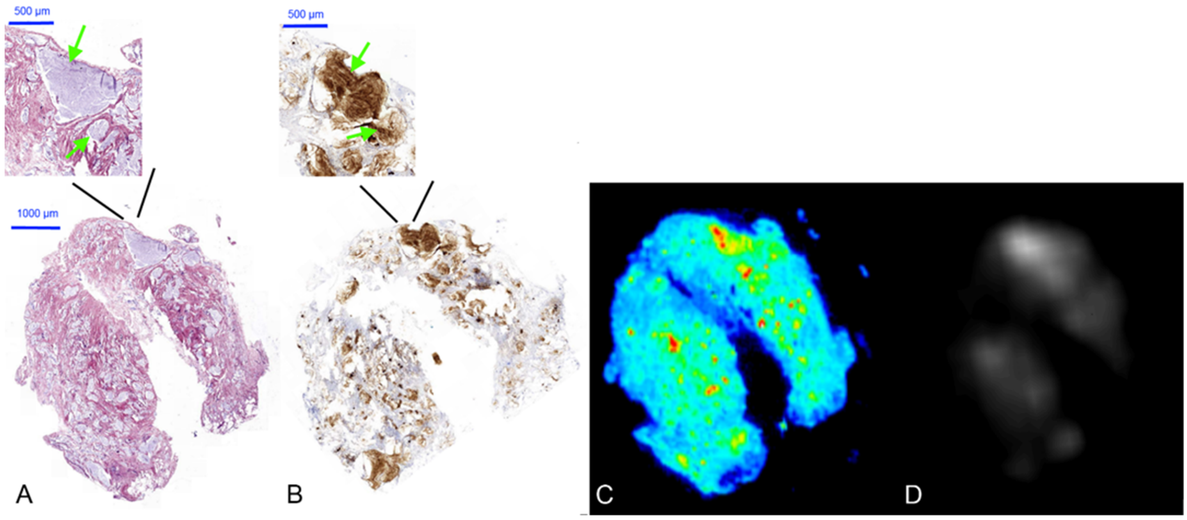

Figure S3b: Tumor with mucin pools (other patient than depicted in figure 3a). A. H&E staining and zoom, B. CEA immunohistochemical staining, C. Flatbed fluorescence scan image, D. Autoradiography. Note the tracer accumulation in the mucin pools (green arrows).

© 2020 by the authors. Submitted for possible open access publication under the terms and conditions of the Creative Commons Attribution (CC BY) license (http://creativecommons.org/licenses/by/4.0/).
